# Functional Characterization of the *Frost* Gene in *Drosophila melanogaster*: Importance for Recovery from Chill Coma

**DOI:** 10.1371/journal.pone.0010925

**Published:** 2010-06-02

**Authors:** Hervé Colinet, Siu Fai Lee, Ary Hoffmann

**Affiliations:** 1 Earth and Life Institute, Biodiversity Research Centre, Université catholique de Louvain, Louvain-la-Neuve, Belgium; 2 Centre for Environmental Stress and Adaptation Research, Department of Genetics, Bio21 Institute, The University of Melbourne, Parkville, Victoria, Australia; Michigan State University, United States of America

## Abstract

**Background:**

Almost all animals, including insects, need to adapt to temperature fluctuations. The molecular basis of thermal adaptation is not well understood, although a number of candidate genes have been proposed. However, a functional link between candidate genes and thermal tolerance has rarely been established. The gene *Frost* (*Fst*) was first discovered when *Drosophila* flies were exposed to cold stress, but the biological function(s) of *Fst* has so far not been characterized. Because *Fst* is up-regulated after a cold stress, we tested whether it was essential for chill-coma recovery.

**Methodology/Principal Findings:**

A marked increase in *Fst* expression was detected (by RT-PCR) during recovery from cold stress, peaking at 42-fold after 2 h. The GAL4/UAS system was used to knock down expression of *Fst* and recovery ability was assessed in transgenic adults following 12 h of chill coma at 0°C. The ability to recover from cold stress (short-, medium- and long-term) was significantly altered in the transgenic adults that had *Fst* silenced. These findings show that *Fst* plays an essential role in the recovery from chill coma in both males and females.

**Conclusions/Significance:**

The *Frost* gene is essential for cold tolerance in *Drosophila melanogaster* and may play an important role in thermal adaptation.

## Introduction

Insects subjected to seasonally low temperatures have evolved a range of physiological and molecular adaptations to survive [1]. The molecular mechanisms behind cold stress and associated chilling injuries are complex and still poorly understood [2], [3]. *Drosophila melanogaster* has adapted successfully to diverse thermal environments and provides a useful model system for understanding the molecular basis of thermal adaptation.

While some studies have considered genes that might be involved in cold tolerance in *Drosophila* [4], the molecular basis of cold stress resistance is poorly understood in comparison to heat resistance. It appears that more genes/proteins are activated during recovery phases following cold stress compared to the actual stress period [2], [5] and these phases need to be differentiated in experimental studies [6]. Recovery from chill-coma is a trait widely studied by evolutionary geneticists (e.g. ref [4], [7]) because it is adaptively significant [4], [8] but its underlying molecular basis is not well-understood.

*Frost* (*Fst*) is one of the few candidate genes that have been implicated in cold tolerance in *D. melanogaster*. This gene was first discovered and characterized by Goto [9] in flies exposed to cold stress. Recent studies have also suggested that *Fst* might be a good candidate for thermal adaptation [11], [12]. *Fst* was up-regulated during recovery from cold stress but, unlike heat-shock genes [7], *Fst* expression was not altered after heat stress [10]. However, a functional relationship between *Fst* and cold tolerance remains to be established. *Fst* has also been reported to respond weakly to a range of abiotic stressors, such as dietary shifts, desiccation, chemical toxicity, insecticide exposure and hypoxia [10], [13]–[16]. *Fst* may also be involved in immune response against virus, bacteria and fungi [17]–[20].

In the present study we showed that the mRNA level of *Fst* was markedly increased in adults recovering from cold stress. We demonstrated that silencing *Fst* by transgenic RNA inference impaired the recovery process from chill coma in both sexes. Expression of *Fst* thus seems to be crucial for developing cold tolerance in *D. melanogaster* adults.

## Methods

### *Drosophila* stocks and breeding conditions

The wild type *D. melanogaster* strain was derived from about 50 females collected in Innisfail (Australian east coast) in May 2008 (see ref [7] for more details). RNAi-mediated *Fst* knockdown was achieved using the GAL4/UAS system [21]. The *UAS-Fst* line was obtained from the Vienna Drosophila RNAi Center (transformant ID: KK102049) [22]. The *tubulin-*GAL4 (genotype: w^*^; tubP-GAL4/TM3, Act-GFP JMR2, Ser^1^, provided by Phil Batterham, University of Melbourne) and the *actin5C-*GAL4 (Bloomington Drosophila Stock Center, #4414) lines were used separately to drive the expression of the UAS*-Fst*, both resulted in ubiquitous *Fst* mRNA knockdown. Progeny were tested in cold recovery assays. To control for genetic background effects, the same GAL4 driver lines were crossed to the *w^1118^* line (from BDRC) and their progeny assayed alongside with their GAL4/UAS*-Fst* counterparts. Fly stocks were maintained in 250 ml bottles in uncrowded conditions. Bottles were kept at 25°C, 70% relative humidity, and continuous light on a standard fly medium as previously described [23].

### Cold stress and recovery conditions

All tests were performed using synchronized 4-day old flies, sexed without CO_2_ anaesthesia. To establish the *Fst* mRNA expression during the cold stress and during the recovery period, we used the same method as described in Colinet et al. [7]. Briefly, wild flies were cold stressed at 0°C to induce chill coma, and sampled after 0.25, 3, 6 and 9 h of cold stress (denoted as S025, S3, S6 and S9 respectively). After 9 h of cold stress, flies were allowed to recover at 25°C and *Fst* mRNA expression was measured after 0.5, 2, 4, and 8 h of recovery (denoted as R05, R2, R4 and R8 respectively). For every sampling time there was a corresponding control, consisted of flies kept at 25°C for the same duration (*n* = 4×20 flies).

### RNA extraction and quantitative real time PCRs

RNA extractions were performed using the RNeasy RNA extraction kit and the RNase-Free DNase Set (Qiagen, Australia) as described in Colinet et al. [7]. cDNA was synthesized using the Superscript III First-Strand Synthesis System (Invitrogen, Australia), according to manufacturer's instructions. *Fst* primers were designed with the Primer3 module (http://www.angis.org.au) (forward: 5′-GGAACAGAGGTGGAATAGCCAAAATC-3′ and reverse: 5′-GCCTTGATTGTTTCCGTGAGATTG-3′). The qRT-PCRs were performed on the LightCycler® 480 system (Roche Diagnostics, Australia) following the method previously described [7]. Relative expression ratios (i.e., fold change) were calculated using the 2^−ΔΔCt^ method [24]. *RpS20* was used as a housekeeping reference gene (see ref [7]). To verify the extent of gene knockdown, *Fst* mRNA levels were compared between the untreated flies, kept at 25°C (i.e. basal expression) and the treated flies, recovering for 2 h after cold stress (i.e. during *Fst* up-regulation). Such a comparison in *Fst* expression was conducted separately in males and females (*n* = 3×20 flies per line).

### Chill-coma recovery assays

Three types of assays were used to measure recovery abilities after 12 h of chill-coma at 0°C. Firstly, ‘*short-term recovery*’ was assessed by comparing recovery times of both GAL4/UAS*-Fst* and GAL4*/+* lines at 25°C. Flies were considered recovered when they stood up [25]. Recovery curves were compared between lines using Mantel-Cox analysis with a censoring factor for individuals that did not recover at the end of the experiment. Forty-five flies were monitored for each line. To test for ‘*long-term recovery*’, the mortality of flies after cold stress was assessed when they had been held in food vials at 25°C for 24 h. Chi square contingency tests were used to compare mortality rates between GAL4/UAS*-Fst* and GAL4/+ lines. Mortality rates were based on 150 flies for each line. Finally, an additional ‘*medium-term recovery*’ test was performed with flies derived from *act-*GAL4 crosses. This test was designed to monitor mobility status during 8 h following the cold stress, and represents a modified version of a climbing activity test described elsewhere [26]. Briefly, flies were individually transferred to a 9.5 cm plastic vial. The height flies reached within 7 sec after a mechanical stimulation was noted. Flies were divided into three categories: (a) *injured*, no climbing; (b) *recovering*, slow climbing without reaching the top of the vial within 7 sec; (c) *fit*, fast climbing and reaching the top of the vial within 7 sec. The 7 sec observation time was chosen because preliminary assays showed that all unstressed flies reach the top of a vial within 6 sec (5.1±1.3 sec, *n* = 50). This test was performed repeatedly on the same individuals after 2, 4, 6 and 8 h of recovery (25°C). Flies were maintained on food during this period. Chi square contingency tests were carried out to compare numbers of flies in the three categories for the *act-*GAL4/UAS*-Fst* and *act*-GAL4*/+* lines. Seventy flies were tested for each line. This test was not performed on flies derived from *tub-*GAL4 crosses which were less vigorous even when they were unstressed (34% did not reach the top of the vial within 10 sec, *n* = 50). All statistical tests were performed using Prism V 5.01 (GraphPad software, Inc. 2007).

## Results

**Figure 1:**
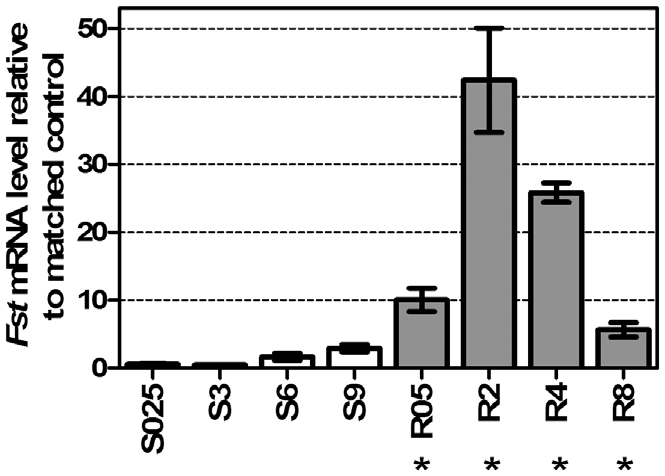
Upregulation of *Fst* during cold stress and recovery. White bars represent cold stressed treatment (S) at 0°C for 0.25 to 9 h and grey bars denote recovery (R) at 25°C for 0.5 to 8 h. Relative expressions are calculated using the 2^−ΔΔCt^ method. Expression levels of *Fst* are normalized against the housekeeping reference *RpS20* and values are expressed as fold change relative to control (mean±SE; *n* = *4*). The symbol (*) indicates when a value is significantly different from untreated controls (*t*-test).

Expression of *Fst* was not altered during the cold stress period, but *Fst* was significantly up-regulated during the recovery phase at 25°C. Expression peaked after 2 h of recovery, when there was a maximal 42-fold change relative to controls (Fig. 1). Because of this significant up-regulation during recovery from cold stress, we suspected that *Fst* may have an essential role in chill-coma recovery.

### Lines derived from *tubulin*-GAL4 driver

**Figure 2:**
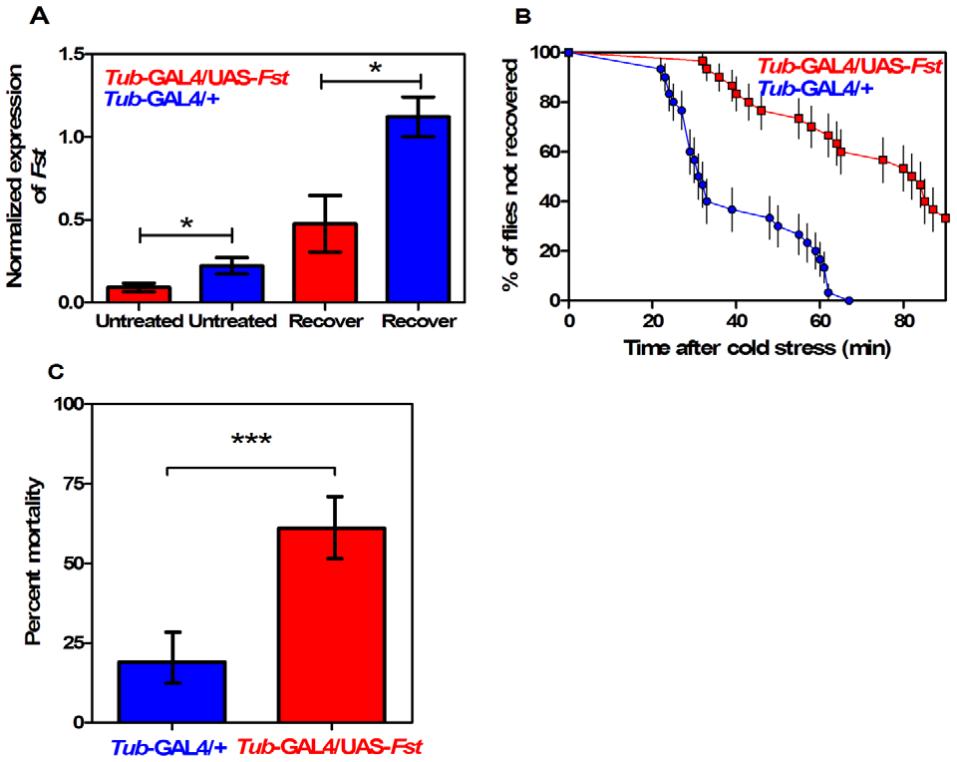
Silencing the cold-inducible *Fst* expression impairs chill coma recovery in *tub*-GAL4-driven females. (A) Expression of *Fst* mRNA in untreated (kept at 25°C) and recovering (2 h at 25°C after 12 h at 0°C) females. Expression levels of *Fst* are normalized against the housekeeping reference *RpS20* and values are √1/x transformed (mean±CI; *n* = 3). The symbol (*) indicates when the level is significantly different in *tub*-GAL4/UAS-*Fst* versus *tub*-GAL4/+ females (*t*-test). (B) Comparison of temporal recovery curves in *tub*-GAL4/UAS-*Fst* (squares) versus *tub*-GAL4/+ (circles) females. Time to recover from chill coma was monitored in females recovering at 25°C after 12 h of cold stress at 0°C. Each dot represents the mean percentage (±SE); 45 females were tested per line. (C) Mortality rate in *tub-*GAL4/UAS-*Fst versus tub*-GAL4/+ females. Mortality was assessed in flies recovering for 24 h at 25°C after 12 h of cold stress at 0°C. Bars represents the percentage (±CI) derived from 150 females in each line. The symbol (*) indicates a significant difference between lines (Chi square test).

**Figure 3:**
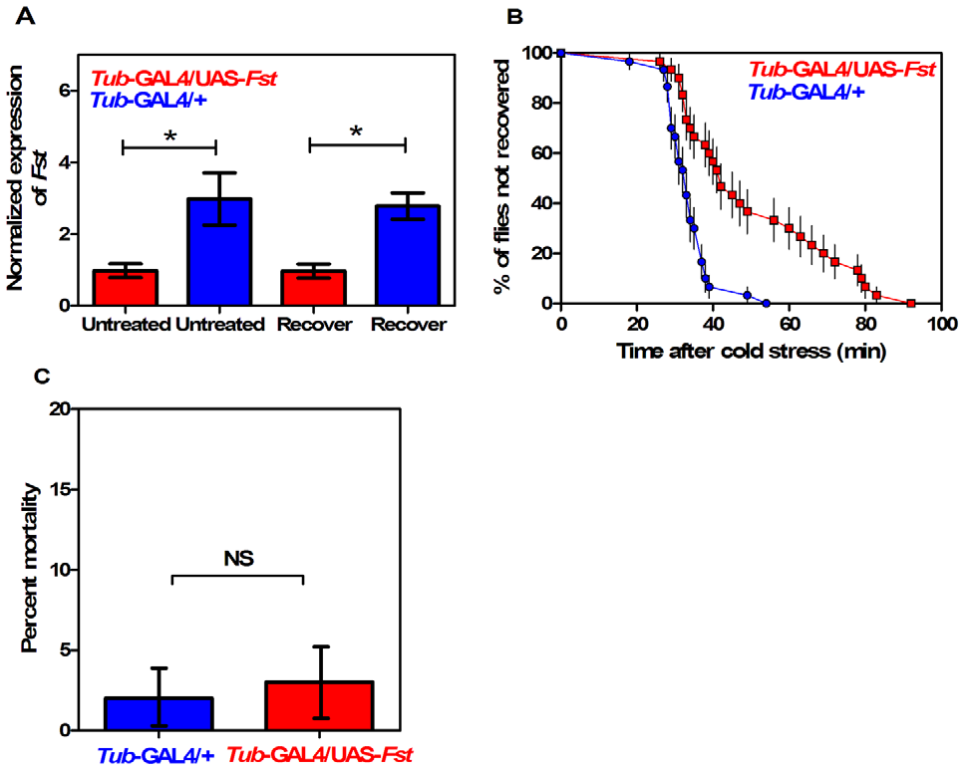
Silencing the cold-inducible *Fst* expression impairs chill coma recovery in *tub*-GAL4-driven males. (A) Expression of *Fst* mRNA in untreated (kept at 25°C) and recovering (2 h at 25°C after 12 h at 0°C) males. Expression levels of *Fst* are normalized against the housekeeping reference *RpS20* and values are √1/x transformed (mean±CI; *n* = 3). The symbol (*) indicates when the level is significantly different in *tub*-GAL4/UAS-*Fst* versus *tub*-GAL4/+ males (*t*-test). (B) Comparison of temporal recovery curves in *tub*-GAL4/UAS-*Fst* (squares) versus *tub*-GAL4/+ (circles) males. Time to recover from chill coma was monitored in males recovering at 25°C after 12 h of cold stress at 0°C. Each dot represents the mean percentage (±SE); 45 males were tested per line. (C) Mortality rate in *tub-*GAL4/UAS-*Fst* versus *tub*-GAL4/+ males. Mortality was assessed in flies recovering for 24 h at 25°C after 12 h of cold stress at 0°C. Bars represents the percentage (±CI) derived from 150 males in each line. The symbol (*) indicates a significant difference between lines (Chi square test).

*Fst* mRNA expression was significantly reduced in *tub-GAL4/UAS-Fst* females compared to *tub-GAL4/+* females, both when flies were untreated (*t* = 10.11, *P*<0.001, IC: 0.166−0.094, *r^2^* = 0.962) and when they were recovering from the cold stress (*t* = 13.36, *P*<0.001, IC: 0.779−0.511, *r^2^* = 0.978) (Fig. 2A). *Fst* expression was also significantly repressed in *tub-GAL4/UAS-Fst* males compared to *tub-GAL4/+* males, both when flies were untreated (*t* = 11.43, *P*<0.001, IC: 2.490−1.517, *r^2^* = 0.970) and recovering from cold stress (*t* = 18.78, *P*<0.001, IC: 2.080 to 1.544, *r^2^* = 0.988) (Fig. 3A). *Fst* knockdown had a significant effect on short-term recovery in both sexes but particularly in females (Fig. 2B, 3B), resulting in significantly different recovery curves (Mantel-Cox: *χ^2^* = 34.33; *df* = 1; *P*<0.001 for females and *χ^2^* = 20.50; *df* = 1; *P*<0.001 for males). In females (Fig. 2B) all the *tub*-GAL4/+ flies recovered within 62 min, while 33% of flies still had not recovered in the *tub-*GAL4/UAS*-Fst* group after 90 min. In males (Fig. 3B) all flies recovered within 90 min but recovery time was longer in the *tub-*GAL4/UAS*-Fst* group. Nevertheless all flies did eventually recover. For the long-term assay, there was a significant difference in mortality between females from the two lines (Fig. 2C) (*χ^2^* = 37.41; *df* = 1; *P*<0.001), with mortality reaching 61% in the *tub-*GAL4/UAS*-Fst* flies compared to 19% in the *tub*-GAL4/+ controls. In males, mortality in the two groups did not differ significantly (*χ^2^* = 0.16, *df* = 1; *P* = 0.68) (Fig. 3C).

### Lines derived from *actin*-GAL4 driver

**Figure 4:**
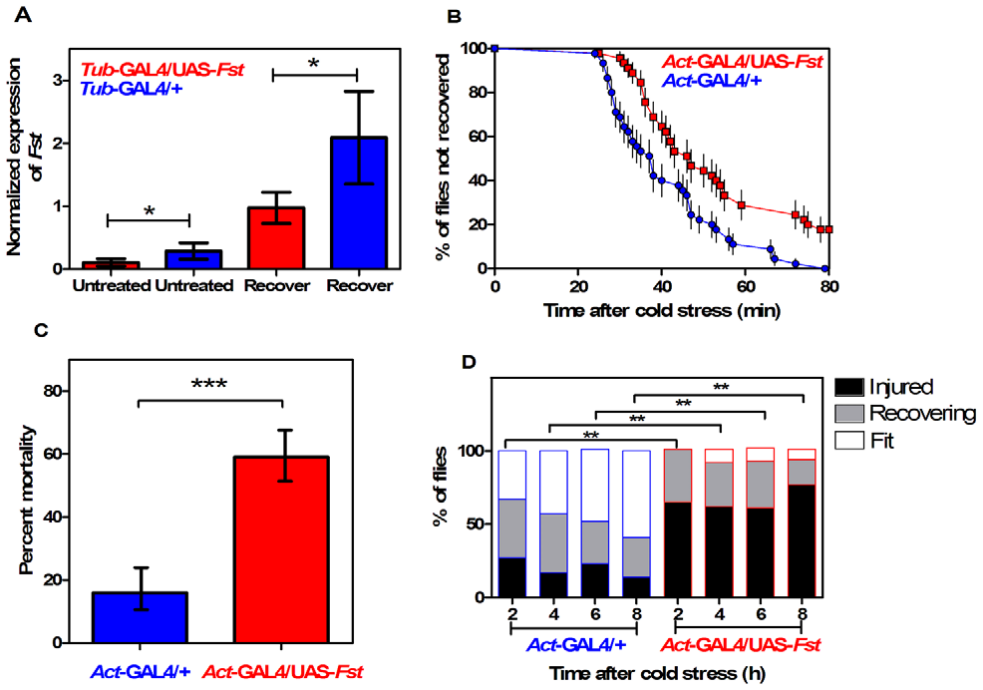
Silencing the cold-inducible *Fst* expression impairs chill coma recovery in *act*-GAL4-driven females. (A) Expression of *Fst* mRNA in untreated (kept at 25°C) and recovering (2 h at 25°C after 12 h at 0°C) females. Expression levels of *Fst* are normalized against the housekeeping reference *RpS20* and values are √1/x transformed (mean±CI; *n* = 3). The symbol (*) indicates when the level is significantly different in *act*-GAL4/UAS-*Fst* versus *act*-GAL4/+ females (*t*-test). (B) Comparison of temporal recovery curves in *act*-GAL4/UAS-*Fst* (squares) versus *act*-GAL4/+ (circles) females. Time to recover from chill coma was monitored in females recovering at 25°C after 12 h of cold stress at 0°C. Each dot represents the mean percentage (±SE); 45 females were tested per line. (C) Mortality rate in *act-*GAL4/UAS-*Fst* versus *act*-GAL4/+ females. Mortality was assessed in flies recovering for 24 h at 25°C after 12 h of cold stress at 0°C. Bars represents the percentage (±CI) derived from 150 females in each line. The symbol (*) indicates a significant difference between lines (Chi square test). (D) Climbing activity monitored in *act-*GAL4/UAS-*Fst* versus *act*-GAL4/+ females. Measurements were taken in recovering females after 2, 4, 6 and 8 h at 25°C following 12 h at 0°C. Flies were categorized as fit (fast climbing) or recovering (slow climbing) or injured (no climbing). The symbol (*) indicate significant differences between lines (Chi square test, *n* = 70).

**Figure 5:**
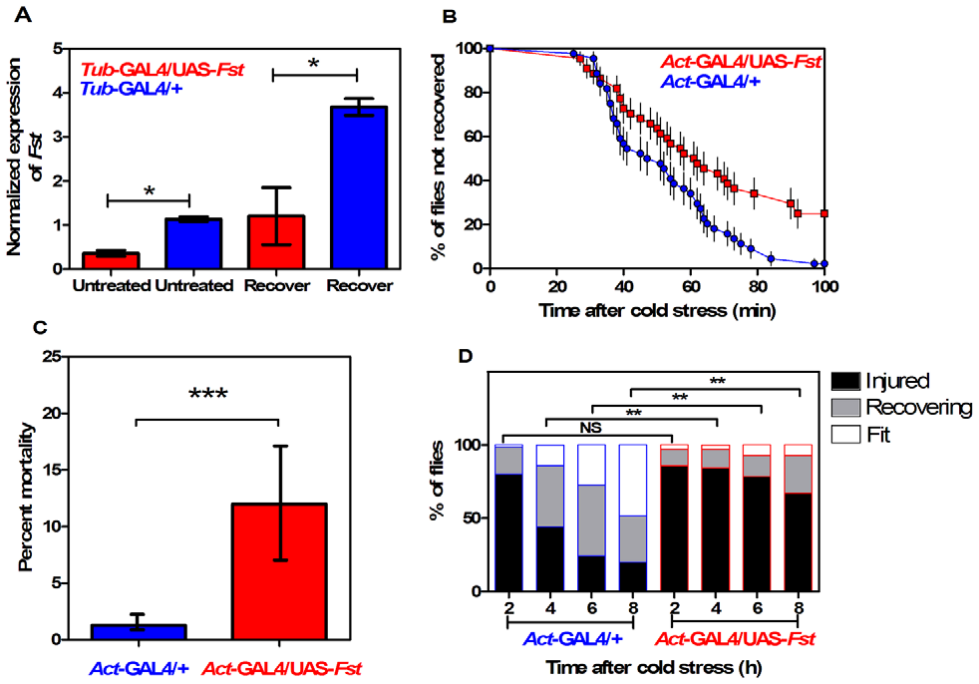
Silencing the cold-inducible *Fst* expression impairs chill coma recovery in *act*-GAL4-driven males. (A) Expression of *Fst* mRNA in untreated (kept at 25°C) and recovering (2 h at 25°C after 12 h at 0°C) males. Expression levels of *Fst* are normalized against the housekeeping reference *RpS20* and values are √1/x transformed (mean±CI; *n* = 3). The symbol (*) indicates when the level is significantly different in *act*-GAL4/UAS-*Fst* versus *act*-GAL4/+ males (*t*-test). (B) Comparison of temporal recovery curves in *act*-GAL4/UAS-*Fst* (squares) versus *act*-GAL4/+ (circles) males. Time to recover from chill coma was monitored in males recovering at 25°C after 12 h of cold stress at 0°C. Each dot represents the mean percentage (±SE); 45 males were tested per line. (C) Mortality rate in *act-*GAL4/UAS-*Fst* versus *act*-GAL4/+ males. Mortality was assessed in flies recovering for 24 h at 25°C after 12 h of cold stress at 0°C. Bars represents the percentage (±CI) derived from 150 males in each line. The symbol (*) indicates a significant difference between lines (Chi square test). (D) Climbing activity monitored in *act-*GAL4/UAS-*Fst* versus *act*-GAL4/+ males. Measurements were taken in recovering males after 2, 4, 6 and 8 h at 25°C following 12 h at 0°C. Flies were categorized as fit (fast climbing) or recovering (slow climbing) or injured (no climbing). The symbol (*) indicate significant differences between lines (Chi square test, *n* = 70).

*Fst* mRNA expression was significantly reduced in *act-GAL4/UAS-Fst* females compared to *act-GAL4/+* females, both when flies were untreated (*t* = 5.47, *P* = 0.005, IC: 0.273−0.089, *r^2^* = 0.882) and when they were recovering from the cold stress (*t* = 6.19, *P* = 0.003, IC: 1.615−0.615, *r^2^* = 0.905) (Fig. 4A). *Fst* expression was also significantly suppressed in *act-GAL4/UAS-Fst* males compared to *act-GAL4/+* males, both when flies were untreated (*t* = 37.60, *P*<0.001, IC: 0.833−0.719, *r^2^* = 0.997) and recovering from the cold stress (*t* = 15.78, *P*<0.001, IC: 2.913−2.041, *r^2^* = 0.984) (Fig. 5A). Short-term recovery was significantly different between lines for both sexes (Fig. 4B, 5B) (Mantel-Cox: *χ^2^* = 12.50; *df* = 1; *P*<0.001 for females; *χ^2^* = 9.63; *df* = 1; *P* = 0.002 for males). For females (Fig. 4B), all the *act-*GAL4/+ control flies recovered within 80 min, while 18% of flies had not recovered in the *act-*GAL4/UAS-*Fst* group. A similar pattern was observed in males (Fig. 5B) with 25% of flies failing to recover in the *act-*GAL4/UAS*-Fst* group after 100 min. All flies eventually recovered. For the long-term recovery assay, a significant difference was observed in females (*χ^2^* = 60.23; *df* = 1; *P*<0.001), mortality reached 59% in the *act-*GAL4/UAS*-Fst* flies compared to 16% in the *act-*GAL4/+ controls (Fig. 4C). Males also differed significantly for mortality (*χ^2^* = 0.13; *df* = 1; *P* = 0.002), which reached 12% in the *act-*GAL4/UAS*-Fst* flies and 1.5% in the *act-*GAL4/+ flies (Fig. 5C). In addition, the medium-term recovery tests revealed significant differences in movement patterns between the *act-*GAL4/UAS*-Fst* and the *act-*GAL4/+ control flies (Fig. 4D, 5D) (*χ^2^* tests: *P*<0.05). A high proportion of females were initially injured in the *act-*GAL4/UAS*-Fst* group and this proportion remained high during the observation period (Fig. 4D). In contrast, females from the *act-*GAL4/+ group gradually recovered, with the proportion of designated as ‘fit’ increasing while ‘injured’ flies decreased in proportion (Fig. 4D). A similar pattern was observed in the males (Fig. 5D) where the *act-*GAL4/+ flies gradually recovered while the majority of the *act-*GAL4/UAS*-Fst* flies remained injured.

## Discussion

*D. melanogaster* is a chill-susceptible species. At 0°C it falls almost instantly into deep chill-coma because of an inability to maintain muscle resting potentials [27]. In addition to this neuromuscular perturbation, chilling injuries accumulate at low temperatures as a result of various physiological dysfunctions (see ref [3] for review). The molecular mechanisms underlying cold stress and recovery from chill-coma are complex and not well understood. Genes involved in heat shock response are known to affect recovery from cold stress in insects [7], [28], [29]. In addition to heat shock genes, the regulation of other genes is presumably important for cold-tolerance. Indeed, multiple genes appear to be up-regulated during recovery from cold stress [30] and *Fst* is among the candidates suspected to play a role in cold tolerance.

However, the functional relationship between *Fst* and cold tolerance has not been established prior to this study. Using transgenic gene silencing techniques, the expression of *Fst* was knocked down. All recovery traits analyzed (i.e. short-, medium- and long-term) were significantly affected in flies where *Fst* expression was suppressed. Our findings thus show that *Fst* plays an important role in chill coma recovery in both sexes. This is the first time, to our knowledge, that a biological function has been demonstrated for *Fst*. QTL and microarrays studies have suggested that *Fst* might be a candidate for thermal adaptation [11], [12] and our findings indicate that this gene is indeed important for cold recovery.

Although the mechanistic details of how *Fst* functions as a protein have not been resolved, the primary sequence of *Fst* suggests that it resembles a mucin-like protein. Frost contains multiple tandem repeats rich in serine, threonine and proline [9], a typical feature of mucins [31]. Like secreted mucins, Frost contains an 18-amino acid signal peptide at the N-terminus [9]. A homology search in annotated protein database (http://www.geneontology.org/) identified two *D. melanogaster* mucins: Mur18B and Muc11A. *Fst* mRNA is highly enriched in adult malpighian tubule and midgut [32], [33]. Similarly, *Mur18B* and *Muc11A* transcripts are enriched in the tubule of adult flies [34]. The function of insects mucin-like proteins are currently poorly characterized [34] and the relationship between mucins and protection from abiotic stress has not been firmly established. A *Drosophila* mucin gene (*Muc68Ca*) was suggested to play an undefined role in heat shock response [35]. There is evidence that mucins protect from oxidative stress [36], [37], which is a typical feature of chilling-injury [38]. Mucins also provide a physical barrier to cells against pathogens and allow homeostasis of local molecular environments with respect to hydration, ionic composition and concentration [31], [39]. This mucin function may be critical because perturbation of ion homeostasis is directly linked to chilling injuries [40], [41] and its reestablishment occurs during recovery [42]. Among the genes up-regulated during cold stress recovery, many encode membrane-related proteins [30]. This is not surprising since the cell membrane is a primary site of chilling or cold-shock injury, as a result of damage to intracellular organelles and the leakage of ions and other solutes across cell membranes [43], [44]. The *Fst* gene product, presumably a mucin-like protein, may help protect membrane integrity and hence recovery from cold [1]. Silencing *Fst* might thus impair some protective functions against oxidative stress and/or alter aspects of osmoregulation across membranes in the tubule and midgut. Taken together, this study provides evidence that *Fst* is essential for chill-coma recovery in adult *D. melanogaster* and highlights the need to further examine this gene from evolutionary and mechanistic perspectives.
